# Zopiclone-associated hallucinations: a case report and database pharmacovigilance study

**DOI:** 10.3389/fpsyt.2026.1925410

**Published:** 2026-09-16

**Authors:** Pan Zhang, Ruihan Zhang, Yuxuan Gao, Chunxiang Wei, Qian Zhao, Qunfang Yang, Junlin Diao

**Affiliations:** 1 Pharmacy Department, Chongqing Mental Health Center, Chongqing, China; 2 Branch fourteen Team Four, College of Basic Medical Science, Army Medical University (Third Military Medical University), Chongqing, China; 3 Department of Pharmacology, College of Pharmacy and Laboratory Medicine, Army Medical University (Third Military Medical University), Chongqing, China

**Keywords:** elderly, hallucination, pharmacovigilance, spontaneous reporting database, zopiclone

## Abstract

**Aims:**

Zopiclone is widely prescribed for insomnia, but the pharmacovigilance reporting signals for hallucinations remain poorly characterized. This study combines a case report and pharmacovigilance data to evaluate the association between zopiclone and hallucinations, compare hallucination subtype signals, and examine factors associated with higher reporting odds.

**Methods:**

We reported a clinical case assessed by the Naranjo scale and analyzed the Canadian Vigilance Adverse Reaction Database (1994-2025) for zopiclone-associated hallucinations. Three disproportionality methods (ROR, PRR, and BCPNN-IC) were used for signal detection. Univariate and multivariate logistic regression were applied to identify factors associated with higher reporting odds.

**Results:**

In the presented case, auditory hallucinations worsened following zopiclone initiation and improved after discontinuation, with a Naranjo score of 4, suggesting a possible temporal association. A total of 58 valid cases of zopiclone-associated hallucinations were included in the database, accounting for 2.41% of all zopiclone-associated adverse events. All three algorithms detected positive disproportionality signals for zopiclone-associated hallucinations, with auditory hallucinations showing a numerically higher point estimate (ROR = 4.71, 95% CI: 2.87-7.72), visual hallucinations (ROR = 3.49, 95% CI: 2.13-5.72), and hallucination (ROR = 2.39, 95% CI: 1.70-3.37). Multivariate logistic regression analysis showed that age ≥ 65 years was independently associated with higher reporting odds of zopiclone-associated hallucinations (adjusted OR = 2.48, 95% CI: 1.42-4.30, P < 0.01). Sex was not independently associated with reporting odds of hallucinations, while BMI was not included in the multivariable model because of substantial missing data.

**Conclusion:**

Zopiclone is associated with a reporting signal for hallucinations, with auditory hallucinations showing a numerically higher point estimate in this dataset. Age ≥ 65 years was independently associated with approximately 2.5-fold higher reporting odds of zopiclone-associated hallucinations. Caution is warranted when prescribing zopiclone to elderly patients. These findings represent pharmacovigilance signals derived from spontaneous adverse-event reports and do not imply causality. The database contained only one schizophrenia case (n = 1); therefore, no subgroup-specific conclusion can be drawn.

## 1 Introduction

Zopiclone belongs to the non-benzodiazepine Z-hypnotic class and is widely prescribed worldwide for short-term insomnia (1). A considerable proportion of insomniac patients present with concurrent mild-to-severe psychiatric comorbidities, including psychotic and mood disorders (2). Hypnotic treatment in this special population faces unique safety challenges. Hallucination is a rarely reported neuropsychiatric adverse reaction associated with zopiclone (3), and its association with zopiclone remains insufficiently validated by large-scale real-world evidence. Such psychotic symptoms can complicate disease management in patients with psychiatric comorbidities, underscoring the need for systematic pharmacovigilance evaluation of hypnotic safety in this population.

Currently, reports of zopiclone-associated hallucinations are extremely rare, with only sporadic case observations available (4), and existing pharmacovigilance evidence lacks population-based quantitative validation. Prior large-scale pharmacovigilance database analyses calculated only overall hallucination signals and did not stratify auditory and visual subtypes for comparative signal assessment (5). Furthermore, no published work has quantified factors independently associated with higher reporting odds for such events, particularly in elderly patients (6).

To address these gaps, this study adopted a combined design integrating a single clinical case report with disproportionality signal analysis based on the Canadian Vigilance Adverse Reaction Database(CVARD) (7). We selected CVARD because it provides freely downloadable, structured case-level data and has been utilized in previous pharmacovigilance research (8–10), whereas other major databases, such as FAERS (where zopiclone reports are limited due to non-US marketing), VigiBase, and EudraVigilance, either had insufficient relevant data or required formal applications with restricted access that precluded the planned case-level analyses for this study.

We aimed to: (1) quantify and compare the disproportionality reporting signals for zopiclone-associated auditory, visual, and general hallucinations; and (2) identify clinical factors associated with higher reporting odds, with a particular focus on elderly patients. This approach, combining an individual case observation with population-wide database mining, constitutes a key feature of our study, offering both clinical reference and real-world pharmacovigilance signals for zopiclone use in clinical practice.

## 2 Case report

A 77-year-old female weighing 50kg presented with a 20-year history of auditory hallucinations, persecutory delusions, and poor sleep. She had been diagnosed with schizophrenia at an external hospital and was discharged after initial improvement, but experienced relapses due to medication self-discontinuation. Six months before the current admission, her symptoms worsened, with suicidal thoughts and behavior. She was admitted to our hospital and treated with risperidone oral solution 1 mg/d. During that hospitalization, she ingested rodenticide and developed poisoning. After treatment at another hospital, she improved but still had psychotic symptoms. She returned to our hospital and received risperidone 1.5 mg at noon and 2 mg at night, leading to symptom improvement, and was discharged. After discharge, she experienced drooling and dizziness and reduced risperidone to 2 mg/day on her own. One month before admission, she reported hearing a man and a woman talking outside, thinking that they were small flying people who monitored and tracked her and could read her thoughts. Three days before admission, she reported seeing a black shadow and hearing someone talking to her, saying that she had committed murder. She left home to rent a house to hide from perceived persecutors. Her family was concerned about the worsening of the patient’s condition and brought her to our hospital on 27 January 2026.

Her past medical history included a 30-year history of hand tremor treated with long-term trihexyphenidyl, grade 3 hypertension (very high risk) controlled with amlodipine besylate 2.5 mg once daily, and lacunar cerebral infarction diagnosed six months previously treated with conventional Chinese medicines (Naomaitai capsules, Ginkgo biloba tablets and Qiangli Dingxuan tablets). She had undergone bilateral glaucoma surgery 18 years earlier and total thyroidectomy 11 years earlier, the latter complicated by permanent hypothyroidism for which she had been on irregular levothyroxine replacement.

On admission, she was given amlodipine besylate 2.5 mg once daily, aspirin 100 mg once daily, Naomaitai capsules 1 g three times daily, trihexyphenidyl 2 mg at night, levothyroxine 50 μg daily, and risperidone 2 mg at night. On day 1 after admission, the blood concentration of risperidone was 13.37 ng/ml, and paliperidone was 19.83 ng/ml. On day 2, risperidone was increased to 1 mg at noon and night dose unchanged. On day 7, she reported no visual shadows and auditory hallucinations persisted but were less frequent and unclear. She had no delusions. The blood concentration of risperidone was 12.98 ng/ml and paliperidone was 34.95 ng/ml. On day 9, risperidone was increased to 1.5 mg in the morning and 2 mg at night. On day 30, she reported that auditory hallucinations had decreased and were difficult to hear clearly. She could ignore them without significant distress, but her sleep was poor. Zopiclone 7.5 mg at night was added. The blood concentration of risperidone was 23.29 ng/ml and paliperidone was 66.12 ng/ml. On day 35, she reported improved sleep but increased auditory hallucinations. Risperidone was adjusted to 4 mg/day. On day 42 she still complained of frequent auditory hallucinations without external stimuli, and quetiapine 25 mg at night was added. On day 45, auditory hallucinations did not improve, but sleep was adequate. Zopiclone was discontinued. On day 46, auditory hallucinations improved compared to before. On day 49, she reported that voices had decreased and she recognized them as “not real”. Her diet and sleep were normal. The Naranjo Adverse Drug Reaction Probability Scale yielded a score of 4, indicating a possible temporal relationship between zopiclone and the exacerbation of auditory hallucinations (**Supplementary Table S1**).

Interpretation of this case is complicated by multiple concurrent confounding factors, including fluctuating psychotic symptoms, antipsychotic dose adjustments, initiation of quetiapine, chronic trihexyphenidyl use, cerebrovascular disease, and hypothyroidism. The Naranjo score of 4 supports only a possible association; definitive causal attribution cannot be drawn from this single case. Further discussion of these confounders is provided in the discussion section.

## 3 Methods

### 3.1 Data source and study design

The raw data of adverse reactions in this study were obtained from the CVARD pharmacovigilance spontaneous reporting database, extracted from January 1994 to December 2025. Inclusion criteria: (1) Zopiclone listed as the primary suspected drug; (2) The preferred terminology for preferred term (PT) includes Hallucination (10019063), Hallucination visual (10019075), Hallucination auditory (10019070), Hallucination gustatory (10019071), Hallucination olfactory (10019072), Hallucination tactile (10019074), Hallucination synesthetic (10062824), Somatic hallucination (10062684), Paroxysmal perceptual alteration (10063117), Fever hallucinations (10088916), Hallucinations mixed (10019079). The PT terms listed above represent the full search strategy. Subtypes with no retrievable cases were not included in the final analysis. Cases were excluded if the primary suspected drug was not zopiclone or if the adverse event was unrelated to hallucinations.

### 3.2 Signal detection

Three classic disproportionality methods, ROR, PRR, and BCPNN-IC, were used to mine adverse drug reaction (ADR) signals. The calculation formulas and positive criteria for each algorithm are shown in **Supplementary Table S2**.

### 3.3 Subgroup analysis and logistic regression

Among all adverse-event reports with zopiclone as the primary suspected drug, 2×2 contingency tables (**Supplementary Table S3**) were constructed to compare reporting signals across different age groups (<65 years vs. ≥ 65 years), sex, BMI (<25 kg/m² vs. ≥ 25 kg/m²), and subgroups with schizophrenia comorbidity. The subgroup ROR, 95% confidence interval (CI), and P-value were calculated.

Logistic regression was performed on all adverse-event reports where zopiclone was listed as the primary suspected drug (total N = 2405). The binary dependent variable was defined at the report level: a report was coded 1 if it contained at least one of the pre-specified hallucination-related MedDRA PTs, and coded 0 if no hallucination-related PT was present; reports without hallucination-related PTs served as the control group. Candidate independent variables included age group (<65 years vs. ≥ 65 years), sex, BMI (<25 kg/m² vs. ≥ 25 kg/m²). Schizophrenia comorbidity was considered as a candidate variable, but as only one report documented schizophrenia comorbidity among the hallucination cases, it could not be incorporated into the regression model and was only described narratively.

Univariate logistic regression was performed for all three candidate independent variables. For the primary multivariate logistic regression model, only age group and sex were included as covariates. BMI was excluded from the multivariate model due to its very high rate of missing observations and was assessed only in univariate analysis. Only reports (n = 2300) with non-missing values for age and sex were retained for modelling, and no data imputation was performed. No pre-specified sensitivity analyses were undertaken due to severe data sparsity for key variables. All tests were two-sided, with P < 0.05 indicating statistical significance.

Logistic regression based on spontaneous adverse-event reports estimates reporting odds rather than population-level clinical risk, and these estimates are subject to the inherent reporting bias of pharmacovigilance databases.

### 3.4 Latency analysis

For cases (n = 14) with documented exact onset dates of hallucination occurrence, the median latency and IQR were calculated, and the Weibull distribution was used to fit the cumulative incidence curve.

All statistical analyses were conducted using Python 3.10.12 with relevant libraries (pandas, numpy, scipy, statsmodels, and lifelines).

## 4 Results

### 4.1 Baseline characteristics

A total of 58 valid cases of zopiclone-associated hallucinations were included, accounting for 2.41% of all zopiclone-associated adverse events. The sex distribution was balanced, with 28 males and 28 females. The largest age group was 75-89 years, accounting for 25.86%, followed by 45-59 years old, accounting for 22.41%. Age data were missing in 15.52% of cases, and body weight data were missing in 79.31%, which limits obesity-related analysis. The number of reports varied across reporting periods, with the highest proportion observed from 2016 to 2020 (31.03%), followed by a decrease in 2021–2025 (18.97%). **Table 1** summarizes the baseline characteristics of the study population, and **Figure 1** provides a more intuitive representation of the baseline demographic distribution of zopiclone-associated adverse events.

**Table 1:** Baseline characteristics of the study population.

Characteristics		n	%
Sex	Male	28	48.28
	Female	28	48.28
	Missing	2	3.45
Weight (kg)	60-74	9	15.52
	75-89	2	3.45
	90-119	1	1.72
	Missing	46	79.31
Age (years)	0-17	3	5.17
	18-44	10	17.24
	45-59	13	22.41
	60-74	8	13.79
	75-89	15	25.86
	Missing	9	15.52
Year	1994-2000	3	5.17
	2001-2005	6	10.34
	2006-2010	10	17.24
	2011-2015	10	17.24
	2016-2020	18	31.03
	2021-2025	11	18.97

**Figure 1:**
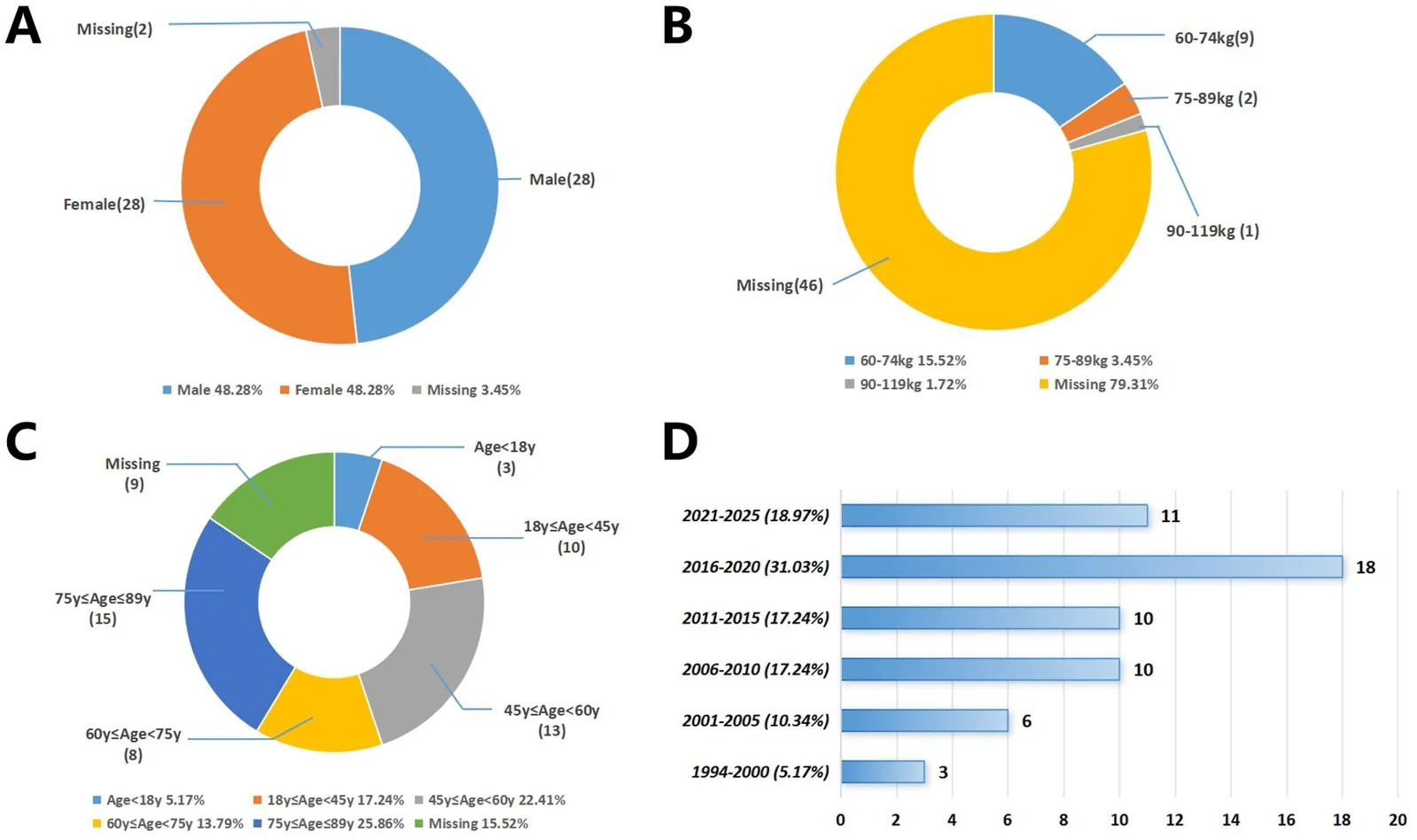
Demographic characteristics of patients in zopiclone-associated adverse events **(A)** sex distribution; **(B)** Weight distribution; **(C)** Age distribution; **(D)** The distribution over reporting years.

### 4.2 Signal detection results

Among all 58 spontaneous reports with zopiclone-associated hallucination events, multiple PT terms could be tagged within a single case. 33 reports were coded under the broad general PT term Hallucination (including unspecified hallucination cases). Separately, 16 reports were labeled with PT Hallucination, Auditory and another 16 with PT Hallucination, Visual. Seven cases carried both auditory and visual hallucination PT labels and were counted as overlapping events in subtype statistics. The remaining terms (including taste hallucinations, smell hallucinations, bodily hallucinations, tactile hallucinations, mixed hallucinations, febrile hallucinations, etc.) did not have relevant cases retrieved or did not meet the positive judgment criteria, therefore they were not included in further analysis. The three algorithms consistently detected positive signals of zopiclone-associated hallucinations, as shown in **Table 2**. Among the hallucination subtypes with detectable signals, auditory hallucinations showed a numerically higher point estimate (ROR = 4.71, 95% CI: 2.87-7.72) than visual hallucinations (ROR = 3.49, 95% CI: 2.13-5.72). However, due to the limited number of reports (n = 16 for each subtype), overlapping cases (n = 7), and largely overlapping 95% confidence intervals, no formal statistical comparison between ROR values was performed. Therefore, this numerical difference is descriptive and does not indicate a statistically or clinically stronger association for auditory hallucinations.

**Table 2:** Signal detection results for zopiclone-associated hallucinations by subtype.

pt	System organ class	n	ROR (95% CI)	PRR (χ²)	IC (IC_025_)
HALLUCINATION	Psychiatric disorders	33	2.39 (1.70-3.37)	2.39 (26.41)	1.25 (0.69)
HALLUCINATION, VISUAL	Psychiatric disorders	16	3.49 (2.13-5.72)	3.49 (28.14)	1.79 (0.89)
HALLUCINATION, AUDITORY	Psychiatric disorders	16	4.71 (2.87-7.72)	4.71 (46.02)	2.22 (1.23)

Signal estimates are derived from 58 hallucination cases. Auditory and visual hallucination PT labels overlapped in 7 individual reports. Largely overlapping 95% confidence intervals reflect the limited case numbers; the numerical rank order of ROR point estimates should be interpreted descriptively, not as evidence of subtype dominance or statistically stronger association. Only PT subtypes that achieved positive disproportionality signal thresholds are presented. ROR, Reporting Odds Ratio; PRR, Proportional Reporting Ratio; IC, Information Component; IC_025_, lower bound of the 95% confidence interval of the IC; PT, Preferred Term; CI, confidence interval, χ², chi-square.

### 4.3 Results of univariate and multivariate logistic regression analysis

Univariate logistic regression was performed across the full set of zopiclone-suspected adverse-event reports (N = 2405). Age ≥ 65 years was associated with higher reporting odds for hallucinations (crude OR=2.39, 95% CI: 1.39-4.13, P < 0.01), whereas sex (crude OR=1.51, 95% CI: 0.89-2.55, P=0.13) and BMI ≥ 25 kg/m^2^ (crude OR=0.49, 95% CI: 0.12-2.01, P=0.32) showed no significant association (**Supplementary Table S4**). For the primary multivariate logistic regression model adjusted for age group and sex, records with missing age or sex were excluded. The final effective analytical sample for the multivariate model comprised 2300 reports. After adjustment for sex, age ≥ 65 years was independently associated with higher reporting odds for zopiclone-associated hallucinations (adjusted OR=2.48, 95% CI: 1.42-4.30, P < 0.01). Sex showed no statistically significant association (adjusted OR=1.65, 95% CI: 0.97-2.80, P=0.07) (**Table 3**). BMI was not included in the multivariate model due to substantial missing data (87.48%) and was evaluated only in univariate analysis.

**Table 3:** Multivariate logistic regression model for hallucinations.

Variable	Number of cases	Adjusted OR (95% CI)	P-value
Sex			
Female	1396	1.00 (Reference)	
Male	904	1.65 (0.97-2.80)	0.07
Age (years)			
<65	1934	1.00 (Reference)	
≥65	471	2.48 (1.42-4.30)	< 0.01

The total pool of zopiclone-associated adverse-event reports was N = 2405. Multivariate logistic regression was performed on complete-case records with non-missing age and sex (n = 2300). BMI was excluded from multivariate analysis due to substantial missing data (87.48%) and was only assessed in univariate logistic regression (presented in **Supplementary Table S4**).

### 4.4 Latency analysis

Among the 14 cases with documented exact onset dates, the observed median latency was 16.5 days (IQR: 10.0-25.0 days), although the temporal trend was not significant (Weibull shape parameter β = 0.48, 95% CI: 0.04-1.26; **Supplementary Figure S1**). Given the limited sample size (n = 14), this analysis is purely descriptive and does not support specific clinical monitoring time-points.

## 5 Discussion

This study, based on the CVARD spontaneous report database (58 zopiclone-associated hallucination cases) combined with a detailed case report of a 77-year-old schizophrenia patient, systematically evaluated the association between zopiclone and hallucinations. The signal detection analysis revealed positive disproportionality signals for general hallucinations, auditory hallucinations and visual hallucinations, with auditory hallucinations showing a numerically higher point-estimate of reporting odds within this dataset. Multivariate regression suggested age ≥ 65 years as a factor independently associated with higher reporting odds. Sex and BMI showed no independent association. However, the latency analysis did not demonstrate a statistically significant temporal trend.

In the presented case, there was a temporal association between zopiclone exposure and the emergence of aggravated hallucinatory symptoms, with symptom improvement observed following zopiclone discontinuation. However, multiple concurrent confounders, including fluctuating symptoms of underlying schizophrenia, escalation of risperidone, initiation of quetiapine, chronic trihexyphenidyl use, cerebrovascular lesions, and hypothyroidism, complicated interpretation. Notably, symptom improvement temporally overlapped with intensified antipsychotic treatment, making it difficult to attribute the change solely to zopiclone withdrawal. The Naranjo score of 4 indicates only a possible contribution of zopiclone; definite causal inference cannot be drawn based on this single observation.

Published observations regarding zopiclone-associated hallucinations have largely stemmed from isolated case reports (11). Studies using pharmacovigilance databases have detected positive disproportionality signals for hallucinations associated with Z-class drugs. However, they have not quantitatively analyzed relevant factors associated with higher reporting odds (12). In addition, this study combines a case report with large-sample database analysis, offering complementary perspectives from individual clinical observation and population-based pharmacovigilance analysis.

The exact mechanism underlying zopiclone-associated hallucinations remains incompletely elucidated. Several plausible pathophysiological hypotheses have been proposed, including GABAergic system imbalance (13, 14), disrupted sleep-wake transition (15–19), and impaired sensory gating (20). However, these mechanistic explanations remain speculative and are not directly validated by the current pharmacovigilance data and case observation. Further experimental and clinical investigations are required to confirm these potential pathways. Similar hallucinatory phenomena have been reported with other Z-class drugs (12, 21–23).

Elderly patients may be a key susceptible population for zopiclone-associated hallucinations. Older adults exhibit heightened sensitivity to Z-drug adverse effects with more pronounced reactions at equivalent doses, supporting the general recommendation for reduced dosing in this age group (24–26). This increased susceptibility may be attributed to reduced drug clearance, age-related changes in the blood-brain barrier and neurotransmitter function (27, 28), cognitive and psychomotor impairment, concurrent cerebrovascular or neurodegenerative disorders, and polypharmacy (29). Collectively, these factors may lower the threshold for hallucinatory events, rendering elderly individuals more vulnerable to central nervous system effects of standard-dose zopiclone.

In addition, dosage magnitude, plasma drug concentration, and concurrent drug interactions may influence the reporting of zopiclone-associated hallucinations. Z-drug adverse events are generally dose-dependent, and co-administration with other central nervous system depressants can exacerbate sedation, confusion, and cognitive dysfunction, potentially increasing the likelihood of severe neurological or respiratory compromise (22, 30). Therefore, cautious clinical assessment for hallucinatory events is warranted in patients concurrently receiving antipsychotics, antidepressants, benzodiazepines, opioids, antihistamines, or alcohol (31).

This study has several limitations. Spontaneous reporting databases are subject to underreporting, selective reporting, and missing data, limiting causal inference (32, 33). The BMI missing rate was 87.48%, precluding meaningful analysis of this variable. Dosage, treatment duration, and concomitant medication data were unavailable, preventing dose-response and interaction analyses. Latency analysis was limited to 14 of 58 cases with documented exact onset dates, compromising the precision of temporal estimates and precluding definitive conclusions about onset timing. The absence of user denominator data prevented incidence rate calculation. In addition, the index case had multiple comorbidities, limiting generalizability to healthier populations. Finally, only one report involving a patient with schizophrenia was retrieved from the pharmacovigilance database, precluding meaningful subgroup-level analyses. Although our separate clinical index case raises potential considerations for this patient group, similar observations have been reported in other case reports of non-benzodiazepine-hypnotic-related hallucinations (34, 35). Nevertheless, no mechanistic inference (e.g., sensory gating deficits) can be supported by our dataset. This observation remains strictly hypothesis-generating and requires independent validation in larger cohorts enriched for schizophrenia.

## 6 Conclusion

In summary, this study combines large-scale pharmacovigilance data with a detailed case report, suggesting a reporting-level association between zopiclone and hallucinations. Auditory hallucinations showed a numerically higher point-estimate of reporting odds within this dataset, and age ≥ 65 years was identified as a factor independently associated with higher reporting odds (adjusted OR = 2.48). Clinicians should remain vigilant for hallucinatory adverse events, particularly when initiating treatment in elderly patients. Notably, findings derived from spontaneous reporting data cannot establish causal relationships.

## Data Availability

The raw data supporting the conclusions of this article will be made available by the authors, without undue reservation.
