# Supplementary material for: Zopiclone-associated hallucinations: a case report and database pharmacovigilance study
Source: Front Psychiatry. 2026 Sep 16;17:1925410. doi: 10.3389/fpsyt.2026.1925410 (PMC13624146; doi:10.3389/fpsyt.2026.1925410)
Supplement: Supplementary file 1 [file Supplementaryfile1.docx]

Supplementary Table S1. The Naranjo scale assessment

| **Question** | **Score** | **Justification** |
| --- | --- | --- |
| 1. Previous conclusive reports? | +1 | Previous literature has reported that zopiclone can cause hallucinations[4] |
| 2. Did ADR occur after suspect drug? | +2 | Within 5 days (Day 35) of zopiclone initiation, there was an exacerbation of auditory hallucinations |
| 3. Did ADR improve after drug withdrawal? | +1 | After discontinuing Zopiclone, the auditory hallucinations improved on Day 46 and further improved on Day 49 |
| 4. Did ADR reappear on re-exposure? | 0 | Not attempted |
| 5. Are there alternative causes? | 0 | Concurrent antipsychotic dose escalation (risperidone doubled to 4 mg and quetiapine 25 mg added), underlying schizophrenia fluctuation, chronic trihexyphenidyl use, and lacunar infarction overlap temporally with zopiclone withdrawal |
| 6. Did placebo cause similar reaction? | 0 | Not applicable |
| 7. Was drug concentration at toxic level? | 0 | Not measured |
| 8. Was response dose-dependent? | 0 | Not applicable |
| 9. Previous ADR to same drug class? | 0 | Not previously used |
| 10. Was ADR confirmed by objective evidence? | 0 | Relying on subjective reports from patients without independent objective evidence. |
| **Total Score** | **+4** | **Possible (score 1–4)** |

Supplementary Table S2. Four algorithms used for signal detection

| **Algorithms** | **Equation** | **Criteria** |
| --- | --- | --- |
| ROR |  | lower limit of 95%CI >1, N≥3 |
|  |  |  |
| PRR |  | PRR≥2, χ^2^≥4, N≥3 |
|  |  |  |
| BCPNN |  | IC₀₂₅ > 0 |
|  |  |  |

Supplementary Table S3. 2×2 contingency table

|  | **Target AEs** | **Non-target AEs** | **Total** |
| --- | --- | --- | --- |
| Target drugs | a | b | a+b |
| Non-target drugs | c | d | c+d |
| Total | a+c | b+d | N=a+b+c+d |

Supplementary Table S4. Univariate logistic regression model for hallucinations

| **Variable** | **Number of cases** | **Crude OR (95% CI)** | **P-value** |
| --- | --- | --- | --- |
| sex |  |  |  |
| Female | 1396 | 1.00 (Reference) |  |
| Male | 904 | 1.51 (0.89-2.55) | 0.13 |
| Age(years) |  |  |  |
| <65 | 1934 | 1.00 (Reference) |  |
| ≥65 | 471 | 2.39(1.39-4.13) | ＜ 0.01 |
| BMI(kg/m²) |  |  |  |
| <25 | 151 | 1.00 (Reference) |  |
| ≥25 | 150 | 0.49 (0.12-2.01) | 0.32 |

Note：Univariate logistic regression was performed separately for each variable. BMI analysis was restricted to the 301 reports with available BMI data

Supplementary Figure S1. Weibull distribution of latency period from zopiclone administration to the occurrence of hallucinatory adverse reactions.
